# Ultrathin‐Film Titania Photocatalyst on Nanocavity for CO_2_ Reduction with Boosted Catalytic Efficiencies

**DOI:** 10.1002/gch2.201800032

**Published:** 2018-09-19

**Authors:** Haomin Song, Wei Wu, Jian‐Wei Liang, Partha Maity, Yuying Shu, Nam Sun Wang, Omar F. Mohammed, Boon S. Ooi, Qiaoqiang Gan, Dongxia Liu

**Affiliations:** ^1^ Department of Electrical Engineering The State University of New York at Buffalo Buffalo NY 14260 USA; ^2^ Department of Chemical and Biomolecular Engineering University of Maryland College Park MD 20742 USA; ^3^ Department of Electrical Engineering Photonics Lab King Abdullah University of Science and Technology Thuwal 23955 Saudi Arabia; ^4^ Department of Material Science King Abdullah University of Science and Technology Thuwal 23955 Saudi Arabia; ^5^ W. R. Grace and Company 7500 Grace Dr. Columbia MD 21044 USA

**Keywords:** CO_2_ photoreduction, light absorption, nanocavity, photocatalysis, ultrathin‐film titania

## Abstract

Photocatalytic CO_2_ reduction with water to hydrocarbons represents a viable and sustainable process toward greenhouse gas reduction and fuel/chemical production. Development of more efficient catalysts is the key to mitigate the limits in photocatalytic processes. Here, a novel ultrathin‐film photocatalytic light absorber (UFPLA) with TiO_2_ films to design efficient photocatalytic CO_2_ conversion processes is created. The UFPLA structure conquers the intrinsic trade‐off between optical absorption and charge carrier extraction efficiency, that is, a solar absorber should be thick enough to absorb majority of the light allowable by its bandgap but thin enough to allow charge carrier extraction for reactions. The as‐obtained structures significantly improve TiO_2_ photocatalytic activity and selectivity to oxygenated hydrocarbons than the benchmark photocatalyst (Aeroxide P25). Remarkably, UFPLAs with 2‐nm‐thick TiO_2_ films result in hydrocarbon formation rates of 0.967 mmol g^−1^ h^−1^, corresponding to 1145 times higher activity than Aeroxide P25. This observation is confirmed by femtosecond transient absorption spectroscopic experiments where longer charge carrier lifetimes are recorded for the thinner films. The current work demonstrates a powerful strategy to control light absorption and catalysis in CO_2_ conversion and, therefore, creates new and transformative ways of converting solar energy and greenhouse gas to alcohol fuels/chemicals.

## 1 Introduction

The energy crisis and climate change are emerging global challenges for the 21st century. Photocatalytic conversion of carbon dioxide (CO_2_)‐to‐hydrocarbon (CTH) fuels and chemicals, involving simultaneous greenhouse gas reduction, solar energy conversion, and valuable fuel/chemical production, represents a viable and sustainable process to address these challenges.^1^, ^2^, ^3^ Studies on photocatalytic CO_2_ conversion have explored CO_2_ reduction by water (H_2_O) over semiconductor catalysts such as titania (TiO_2_).^4^, ^5^, ^6^ However, the uphill/multielectron transfer reaction nature, short wavelength cutoff, and fast recombination of charge carriers (electrons/holes, e^−^/h^+^) of TiO_2_ catalyst lead to low efficiency in CO_2_ conversion. In the past decades, considerate efforts have been placed on the development of nanostructured TiO_2_‐based catalysts, such as nanosized TiO_2_ particles (nanowires,^7^ nanotubes,^8^ or nanosheets^9^) and molecular‐sized Ti‐oxide sites anchored in porous materials,^10^, ^11^ to mitigate the limits in photocatalytic CTH process. The nanosized TiO_2_ structures provide diffusion path along their geometrical axis, disfavoring the recombination of e^−^/h^+^ and enhancing multielectron transfer for CO_2_ reduction reactions.^12^, ^13^, ^14^ The molecular‐sized Ti‐oxide sites are spatially isolated and tetrahedrally coordinated in porous matrix, resulting in formation of trapped electron (Ti^3+^)–hole (O^−^) pairs upon ultraviolet (UV) irradiation,^11^, ^15^ which are particularly favorable for CTH reactions. An enhancement in activity in CTH reactions over nanostructured TiO_2_‐based catalysts has been observed. But the photoconversion efficiency is still too low to meet practical applications, mainly due to lack of materials with sufficient light absorption.

One promising scheme to enhance the optical absorption without sacrificing the catalytic activity/selectivity in nanostructured catalysts would be to employ light trapping strategies on photocatalysts. Recently, planar thin film interference in lossy ultrathin layers attracted intensive interests.^16^, ^17^ In particular, this mechanism has been employed to enhance the optical absorption of planar ultrathin photocatalytic α‐Fe_2_O_3_ films for efficient water splitting.^17^ By controlling the planar cavity structure, we demonstrated that the optical absorption within 1.5‐nm‐thick Ge film^18^ and MoS_2_ monolayers^19^ can be enhanced significantly, opening the door to high‐efficiency ultrathin‐film energy conversion materials, structures, and devices.^20^ Importantly, this principle is general and can be implemented into photocatalytic systems to boost the photoconversion efficiency. In this work, we will select the ultrathin‐film TiO_2_ system as an example to implement this light trapping mechanism to enhance the optical absorption of ultrathin TiO_2_ films (down to ≈2 nm) and reveal its unique application for super‐efficient photocatalytic CO_2_ reduction. Due to the boosted photogenerated carrier density and efficient carrier transportation within 2–7 nm thick TiO_2_ thin film catalysts, hydrocarbon formation rate up to 0.967 mmol g^−1^ h^−1^ was obtained experimentally.

## 2 Results

### 2.1 Two‐Layered Al/TiO_2_ Cavity System

#### 2.1.1 Light Trapping Effect

**Figure 1:**
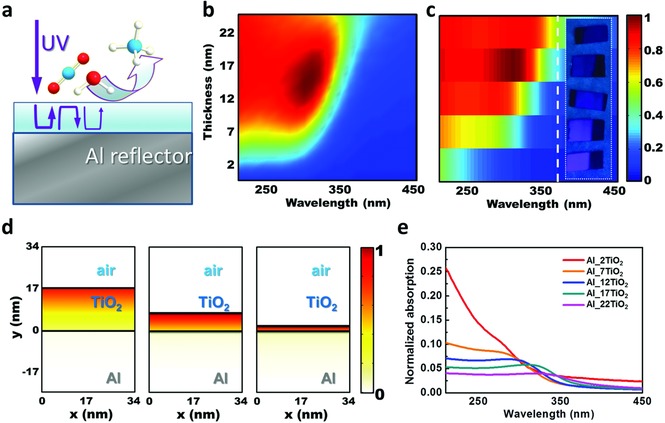
Light trapping within planar nanocavity enhanced ultrathin TiO_2_ films. a) Schematic of a two‐layered UFPLA comprised of sequential photocatalytic/absorptive TiO_2_ thin films and a bottom aluminum (Al) reflector layer. b) Modeled and c) measured total absorption spectra of TiO_2_/Al as a function of the thickness of TiO_2_. d) The spatial absorption distribution in UFPLAs with different TiO_2_ films. e) The measured optical absorption spectra of different TiO_2_ layers normalized by their thicknesses.

To demonstrate the light trapping strategy using planar nanocavities and its spectral tunability, we first developed and analyzed a series of planar structures with controlled thicknesses. As illustrated in **Figure** **1**a, the ultrathin‐film photocatalysis light absorber (UFPLA) is comprised of an aluminum (Al) reflector layer and an absorptive/photocatalytic TiO_2_ ultrathin film on top of the system that can interact with the external environment (i.e., CO_2_ and H_2_O molecules). Details of sample preparation are listed in the Experimental Section. To enable the design of UFPLA, we first employed atomic layer deposition (ALD) to deposit ultrathin TiO_2_ films and characterized their thicknesses and actual optical constants (i.e., refractive index, *n*, and absorption coefficient, *k*; see Figure S1, Supporting Information) using a spectroscopic ellipsometer (Horiba), which is a typical optical method for thin film characterization.^21^, ^22^, ^23^, ^24^ By substituting these optical constants into the numerical modeling package (COMSOL), we then modeled the optical absorption of the TiO_2_/Al planar cavity system (see more details of the modeling in the Experimental Section). In this modeling, the optical absorption of the system at each wavelength from 210 to 450 nm was modeled as the function of the thickness of TiO_2_ films and plotted using the color contour (i.e., the dark blue indicates weak optical absorption and the dark red indicates strong optical absorption). One can see from Figure 1b that strong resonant absorption peaks over 90% from 270 to 325 nm can be obtained in the optimized TiO_2_ film thickness region of 12–19 nm. Beyond this range, the overall absorption will decrease with thinner or thicker TiO_2_ layers. To validate this theoretical prediction, we prepared five ultrathin TiO_2_ films (with the thickness of 2–22 nm) on top of 150‐nm‐thick bare Al films and captured a photograph under the UV light source at 365 nm (see the inset in Figure 1c). One can see that the reflection at 365 nm is lower for thicker TiO_2_ films on Al reflectors. Their measured optical absorption spectra are shown in Figure 1c (original data shown in Figure S2, Supporting Information), confirming the monotonically decreasing reflection at 365 nm (see the white dotted line). One can see that the optimized absorption peak occurred at the thickness of ≈17 nm at the wavelength of ≈310 nm, agreeing well with the theoretical prediction shown in Figure 1b.

#### 2.1.2 Effective Optical Absorption and Volume Carrier Generation

To reveal more details of optical absorption, the spatial absorption distributions at the absorption peak wavelength within 17, 7, and 2 nm TiO_2_ films were modeled using the finite element method (Figure 1d). One can see that the optical absorption within the 17‐nm‐thick film is stronger at the top surface which decreases significantly toward the bottom side; while the optical absorption within thinner films are higher and more uniform along the vertical direction. This mechanism has been implemented in water splitting, using a 26‐nm‐thick α‐Fe_2_O_3_ film on planar Ag‐Au alloy reflector to achieve ≈100% total absorption at the wavelength of 520 nm (i.e., photocatalysis using visible solar energy).^17^ However, in most energy harvesting and conversion applications, there is a well‐known trade‐off between optical absorption and the carrier transportation efficiency, that is, photogenerated carriers cannot be extracted efficiently if the film thickness is larger than the diffusion length of carriers because of carrier recombination. Therefore, a stronger optical absorption may unnecessarily result in an enhanced energy conversion efficiency. Although our reported photocatalysis film thickness with optimized optical absorption here is much thinner than that of materials used in conventional photocatalytic reactions (e.g., 26‐nm‐thick α‐Fe_2_O_3_ film in ref. 17 and 17–22 nm in Figure 1d), the remaining major question is whether they are optimized simultaneously for carrier transportation for the photoreduction of CO_2_. In particular, as shown in Figure 1d, the absorption within the 2‐nm‐thick film is obviously stronger than that in the top 2 nm over the entire 17 nm film. To further reveal this thickness‐dependent carrier density, we normalized the optical absorption spectra with the film thickness (i.e., corresponding to the optical absorption per volume), as shown in Figure 1e. One can see that thinner films generally produced more carriers over a given area, which should be another important factor in addition to the overall optical absorption. These thickness‐dependent photogenerated carrier densities may be strongly related to photocatalytic reactions, which will be discussed in the next section.

#### 2.1.3 Photocatalytic Reduction of CO_2_

**Figure 2:**
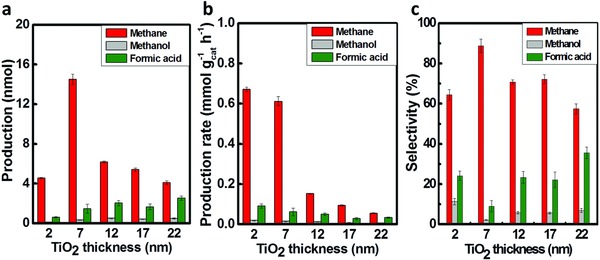
CO_2_ photocatalytic reduction by water on ultrathin TiO_2_ films. a) Measured product type and quantity in each UFPLA sample. b) Product formation rates normalized to per unit mass of TiO_2_ catalyst. c) Product selectivity versus TiO_2_ thin‐film thickness in the UFPLAs.

To demonstrate the photocatalytic performance of these TiO_2_ thin films boosted by UFPLAs, CO_2_ reduction by H_2_O was measured at 298 K (see details in Section S3 and Figure S3, Supporting Information). The product type and quantity from CO_2_ reduction over these five samples after 4 h of Xenon (Xe) light irradiation are plotted in **Figure** **2**a, showing that the major product, methane (CH_4_), accompanied with minor amount of methanol (CH_3_OH) and medium amount of formic acid (HCOOH) were formed. One can see that the sum of CH_4_, CH_3_OH, and HCOOH production varies from 4.8 nmol (4.1 nmol CH_4_ + 0.1 nmol CH_3_OH + 0.6 nmol HCOOH) to 17.6 nmol (14.5 nmol CH_4_ + 0.5 nmol CH_3_OH + 2.6 nmol HCOOH), depending on the thickness of the photocatalytic TiO_2_ films in the ULPFA structure. The most noticeable observation is that the peak product quantity occurred on the sample with a 7‐nm‐thick TiO_2_ film. The higher overall optical absorption of the nanocavity with the 17–22 nm thick TiO_2_ films did not yield the maximum hydrocarbon product formation. Instead, a much weaker optical absorption of the 7‐nm‐TiO_2_/Al sample resulted in ≈2.7‐fold production of CH_4_ than the 17‐nm‐TiO_2_/Al sample (i.e., 5.4 nmol). This discrepancy between product formation and light absorption has not been revealed in other literature, including the recent work using cavity boosted α‐Fe_2_O_3_ thin film for water splitting.^17^ The control experiments were conducted on bare glass and no CO_2_ conversion was observed.

To validate the high efficiency of TiO_2_ thin films in UFPLAs in photocatalytic CO_2_ reduction by water, we employed the state‐of‐the‐art titania catalyst (Aeroxide P25, 0.02 g) to perform the same experiment, and the product formation rate was normalized to unit mass of TiO_2_ catalyst for comparison. The reaction on Aeroxide P25 catalyst produced sole methane product at a rate of 8.44 × 10^−4^ mmol g^−1^ h^−1^. The thin‐film TiO_2_ catalysts in the UFPLAs were all much more active than Aeroxide P25 (Figure 2b), although the activity monotonically decreased with increasing TiO_2_ film thickness on the nanocavities. In particular, 2 and 7‐nm‐thick TiO_2_ films had ≈928 and 816 times higher productivity than that of the Aeroxide P25 catalyst. However, the inconsistency between the unit‐mass productivity of TiO_2_ in UFPLAs in CTH reactions in Figure 2b and the normalized optical absorption shown in Figure 1e indicated that the light absorption is not the sole factor that affects the ultimate photocatalytic reaction efficiency. In addition, Figure 2c shows the hydrocarbon product selectivity varies with TiO_2_ film thickness in the nanocavity structures. The higher film thickness favored formation of oxygenated hydrocarbons. It should be noted that all UFPLA structures enabled methanol, formic acid, and methane formation, in comparison to nearly sole methane formation on Aeroxide P25 catalyst. The product selectivity in photocatalytic CTH reactions is dependent on reactor type, reactant phase, catalyst composition/property, etc.^11^, ^25^, ^26^ Given the fact that all reactions were carried out in the same reactor and reactant mixture, the catalyst composition/property should be responsible for the differences in product selectivity.

#### 2.1.4 Reaction Activity/Selectivity Analysis

**Figure 3:**
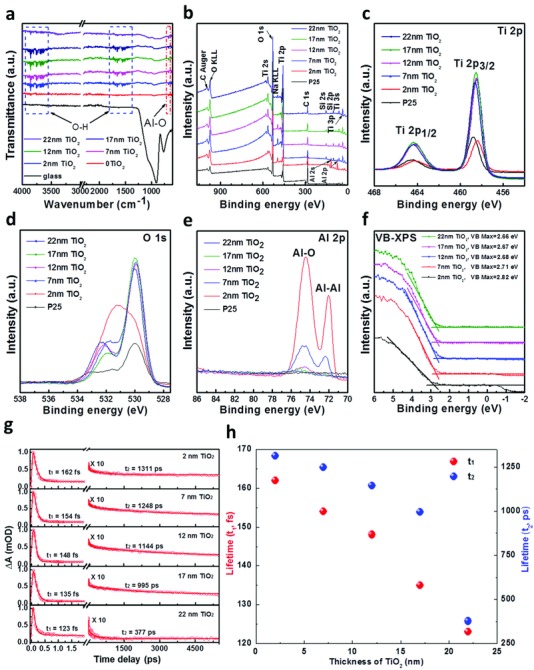
Physicochemical properties of TiO_2_ thin films on planar nanocavity. a) FTIRs and b) XPS spectra of TiO_2_ thin films. c–e) XPS spectra of Ti 2p, O 1s, and Al 2p, respectively, of TiO_2_/Al/glass light absorber structures. f) VBM positions determined from XPS data. g) fs‐TA kinetics traces probed at 860 nm of different thickness of TiO_2_ on glass substrate following 365 nm optical excitation. Solid red line shows exponential fit of the experimental data. h) Lifetime as a function of TiO_2_ thickness.

The activity and selectivity of photocatalytic CO_2_ reduction by water are reported to be significantly affected by properties of the photocatalyst, for example, crystal phase, surface hydroxyl group, defect disorder, electronic structure, etc.^25^ Therefore, a series of characterizations were carried out to understand the physiochemical properties and consequent catalytic performance of the TiO_2_ thin films on the UFPLA structure. Figure S4 (Supporting Information) shows the surface morphology and crystallinity of TiO_2_ generated by ALD process in the UFPLA. The surface of TiO_2_ thin film is uniform, following the pattern of Al layer, independent of the TiO_2_ film thickness (Figure S4a,b, Supporting Information). X‐ray diffraction (XRD) measurement shows that both TiO_2_ and Al are in amorphous form or too small in crystallite sizes to detect since only the diffraction peak of the glass substrate (i.e., 2θ = 22.9°) was observed (Figure S4c, Supporting Information). The Fourier transform infrared spectroscopy (FTIRs) indicated the presence of hydroxyl (—OH) groups in the TiO_2_ thin films, as shown in **Figure** **3**a. All these samples were stored and measured under the same condition. The bending (≈1630 cm^−1^) and stretching (3600–3900 cm^−1^) vibration bands of the OH groups^27^, ^28^ were observed in all samples with TiO_2_ thin films, and the intensity of both bands decreased with TiO_2_ film thickness. It should be noted that the tortuous light trajectory in UFPLAs complicated the characteristic vibration bands of —OH groups. In contrast, the control samples with no TiO_2_ films (i.e., bare glass or Al/glass) did not exhibit as many transmission peaks in the —OH vibrational regions, indicating that the observed signals of UFPLAs were not introduced by H_2_O in the air.

The coordination environment of Ti species in these thin‐film UFPLAs was investigated using X‐ray photoelectron spectroscopy (XPS). Figure 3b shows that the main constituents in the TiO_2_ thin films are titanium, oxygen, aluminum, and the substrate glass (sodium and silicon signals). The XPS spectra of Ti 2p, O 1s, and Al 2p (shown in Figure 3c–e) from the TiO_2_ thin films were examined more closely to analyze their chemical states. Figure 3c illustrates that Aeroxide P25 catalyst has Ti 2p_3/2_ and Ti 2p_1/2_ peaks centered at binding energies of 458.8 and 464.5 eV, respectively. The TiO_2_ thin films in UFPLAs have similar Ti 2p features, but both peaks have lower binding energies than those in Aeroxide P25. The thinner the TiO_2_ film thickness, the larger shift of Ti 2p_3/2_ and Ti 2p_1/2_ peaks toward lower binding energy. The shift in binding energies of Ti 2p core level XPS spectra can be ascribed to two factors: the Ti—O—Al bond formation due to the interaction of TiO_2_ with underlying Al layer^29^, ^30^, ^31^ and the presence of Ti^3+^ species with lower Ti 2p binding energies due to structure defect.^32^, ^33^, ^34^ The thinner TiO_2_ thin films led to more Al—O—Ti bonds and Ti^3+^ species, and thus the Ti 2p_3/2_ and Ti 2p_1/2_ peaks with much lower binding energies. Figure 3d shows O 1s XPS spectra, suggesting oxygen is present in different status in the UFPLA samples. Compared to Aeroxide P25, the emergence of a peak at the binding energy range of 533.2–531.3 eV can be ascribed to oxygen in Al—O bond, Ti^3+^—O bond, and nonlattice oxygen (such as loosely bonded O defects); while the peak (binding energy of 531.3–528.4 eV) existing in Aeroxide P25 and all UFPLAs can be assigned to oxygen in Ti^4+^—O bond. The Al 2p peaks shown in Figure 3e illustrate the presence of aluminum oxide (binding energy of 74.45 eV) and Al metal (binding energy of 72.05 eV) in the TiO_2_ thin‐film catalysts. The distinct peaks in the 2 and 7‐nm‐thick TiO_2_ thin films in Al 2p spectra were due to the usage of X‐ray with in‐depth of 20 nm in the XPS measurement. The formation of Al—O bond might be caused by native oxidation of Al layer, which reveals the presence of very thin spacer in the UFPLAs. From the XPS spectra, we further analyzed the valence band maxima (VBM) of TiO_2_ thin films, as shown in Figure 3f. The shift of VBM to higher energy with decreasing TiO_2_ film thickness is consistent with blueshift of bandgap of TiO_2_ nanostructures compared to that of the bulk catalyst.

Based on analyses above, one can see that the composition and property of TiO_2_ thin films in UFPLAs are distinct from Aeroxide P25 and changes with film thickness, which led to different catalytic activity and selectivity in CTH process. The product selectivity is associated with the chemical states of Ti spices in the catalysts.^15^, ^35^ The higher density of —OH groups in the surface would lead to more hydrophilic nature of the catalyst, and thus the lower opportunity for methanol and formic acid formation.^11^, ^36^ These Ti^3+^ could function as photoinduced electron trapping sites and improved the electron–hole separation rate on the photocatalyst. The lower amount of —OH groups and higher amount of Ti^3+^‐species, structure defects, and large VBM exist in thinner TiO_2_ films in the UFPLAs, which all together contribute to the less formation of oxygenated hydrocarbons compared to thicker films.

The photocatalytic activity is closely correlated to the efficient charge carrier generation/transportation in the UFPLAs, which are reflected by the optical absorption intensity and charge carrier lifetime. The key fundamental question is how many photogenerated carriers can efficiently get involved in the photoreaction with CO_2_ and H_2_O molecules. This is also the most challenging question regarding the carrier dynamics within these ultrathin films, which is largely unexplored. Next, we performed some preliminary analysis to reveal the major mechanism. According to ref. 37, the diffusion length of photo‐generated carriers in TiO_2_ films is very short (e.g., ≈10 nm for holes in n‐TiO_2_ films). Therefore, our hypothesis is that the probability for photogenerated carriers to diffuse to the top surface and participate in the CO_2_ reduction is higher in 2–7 nm thick TiO_2_ films. In thicker samples beyond the effective diffusion lengths, the photogenerated carriers are unlikely to contribute to the chemical reaction. In this scenario, femtosecond transient absorption spectroscopy (fs‐TAS) has been carried out to investigate the carrier dynamics for these TiO_2_ films (see more details of the setup in the Experimental Section). As shown in Figure 3g, we extracted the decay kinetics at 860 nm and plotted them as a function of TiO_2_ thickness. The kinetic traces can be fitted bi‐exponentially with less than 200 fs and ps time constants. The fs and ps time components can be assigned to surface and deep trapped states, respectively.^38^, ^39^ Interestingly, upon increasing the thickness of the TiO_2_, the lifetime of the carriers decreases significantly. As can be clearly seen, decay time changes from 162 fs (fast) and 1311 ps (slow) to 123 fs (fast) and 377 ps (slow), respectively, as the TiO_2_ thickness changes from 2 to 22 nm (as shown in Figure 3h). This would mean that the photogenerated carriers within thinner TiO_2_ films have longer lifetime, indicating the higher probability to flow to the surface and participate in photocatalytic reactions. Consequently, the 2–7 nm thick samples are more efficient in normalized productivity per unit mass (Figure 2b) due to the more efficient volume photogenerated carriers (Figure 1d) and longer carrier lifetime (Figure 3h).

It should be noted that photoluminescence (PL) characterization was also frequently used to reveal the carrier dynamics. However, as we revealed in nanocavity‐manipulated 2D material platforms,^19^ PL intensity and spectral shape from atomically thin semiconductor layers can be changed significantly due to the nanocavity involvement. Therefore, regular PL interpretation cannot be simply implemented here, which is still under investigation. On the other hand, although the unit mass reaction rate of the 2‐nm‐thick TiO_2_/Al system is the best among the studied UFPLAs in Figure 2, the optical absorption of the 2‐nm‐TiO_2_/Al system has not been optimized. Next, we introduced a three‐layered nanocavity system to enhance the optical absorption within 2‐nm‐thick TiO_2_ films.

### 2.2 Three‐Layered Nanocavity with 2‐nm‐TiO_2_ Films

**Figure 4:**
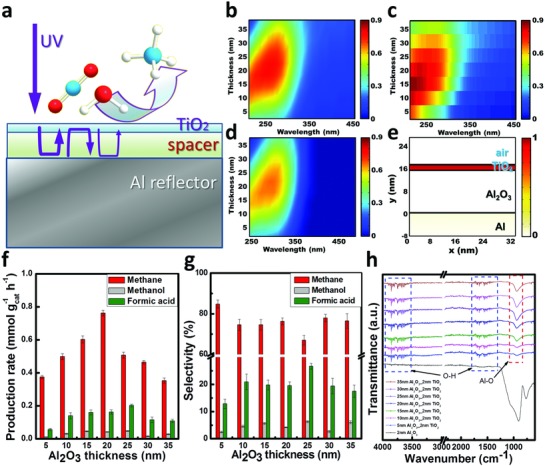
Light trapping within planar nanocavity enhanced ultrathin TiO_2_ films. a) Schematic of a three‐layered UFPLA with an optional spacer layer. b) Measured and c) modeled absorption spectra of Al/Al_2_O_3_/2 nm TiO_2_ as a function of the thickness of Al_2_O_3_. d) Modeled exclusive absorption in TiO_2_ layer as a function of the thickness of TiO_2_. e) The modeled exclusive absorption in the 2‐nm‐thick TiO_2_ layer on 15 nm Al_2_O_3_/Al cavity. The product formation and selectivity are shown in (f) and (g), respectively. h) The FTIRs spectra of the UFPLAs with various Al_2_O_3_ spacer thicknesses.

It is generally believed that significantly enhanced optical absorption within ultrathin films can overcome the long‐existing trade‐off between optical absorption and carrier transportation, which could revolutionize thin‐film energy harvesting and conversion applications (e.g., photocatalysis and photovoltaics). Recently, we proposed a lossless spacer in a three‐layered nanocavity structure to enhance the optical absorption within atomically thin semiconductor layers (e.g., in 1.5‐nm‐thick Ge films,^18^ and monolayers of graphene^40^ and MoS_2_ 19). A suitably designed lossless spacer layer was inserted between the bottom reflector and the top absorptive layers to optimize the disruptive interference condition and therefore enhance the optical absorption. Here, we discuss the potential to enhance the optical absorption of the 2‐nm‐thick TiO_2_ layer using this three‐layered nanocavity structure (**Figure** **4**a). According to the numerical modeling shown in Figure 4b, the peak absorption of the 2‐nm‐thick‐TiO_2_‐Al_2_O_3_‐Al system can be enhanced from ≈30 to ≈90%. To validate this remarkable absorption enhancement, we fabricated another seven samples with different Al_2_O_3_ thickness of 5–35 nm. The measured optical absorption spectra are shown in Figure 4c, agreeing very well with the numerical prediction. When the thickness of the Al_2_O_3_ spacer layer is 15–20 nm, the resonant absorption over 80% can be achieved from 220 to 250 nm. The exclusive absorption in the 2‐nm‐thick TiO_2_ layer is modeled in Figure 4d, confirming the optimized optical absorption obtained with the 15–20 nm thick Al_2_O_3_ spacer layer. Importantly, a much stronger optical absorption was confined within the 2 nm region (Figure 4e). Since the film thickness is identical, the carrier transportation should be similar.

To reveal the optical absorption on the photocatalytic CO_2_ reduction with water, similar photocatalytic CTH reactions were measured at 298 K on the three‐layered nanocavity with 2‐nm‐thick TiO_2_ films and different Al_2_O_3_ thicknesses. As shown in Figure 4f, the hydrocarbon production closely corresponds to the optical absorption. The major product was still methane, mixed with minor amount of methanol and medium amount of formic acid. When the thickness of Al_2_O_3_ layer varied, the CH_4_ production exhibited a semisinusoidal shape at different thickness of spacer layer, which is consistent with the absorption in the TiO_2_ layer. The peak hydrocarbon production was obtained on the nanocavity samples with the Al_2_O_3_ spacer layer of 20 nm. In particular, this sample reached highest hydrocarbon production at 0.967 mmol g^−1^ h^−1^, which is 1145 times higher than the commercial Aeroxide P25 catalyst. In addition, we also obtained an efficient formation of formic acid at the rate of ≈0.2 mmol g^−1^ h^−1^, ≈200 times higher than a recent result produced by 1.66‐nm‐thick TiO_2_ flakes with no nanocavity enhancement (i.e., ≈1.9 μmol g^−1^ h^−1^ 41). The product selectivity in CTH reactions did not show clear trend with Al_2_O_3_ spacer thickness (Figure 4g), which suggests that the property (or thickness) of TiO_2_ thin films in UFPLAs is the dominating factor in influencing product distribution. The same TiO_2_ thickness in these samples hints the same physicochemical properties of UFPLAs. For example, Figure 4h indicates that the increase in Al_2_O_3_ spacer thickness did not enable more —OH groups (bands at ≈1600 and ≈3600 cm^−1^) in the catalyst, while the increase in Al_2_O_3_ spacer thickness was confirmed by the increasing intensity of band at ≈960 cm^−1^.

## 3 Conclusion

In conclusion, the UFPLAs containing TiO_2_ thin film catalysts were developed to target for photocatalytic CO_2_ reduction with water to hydrocarbon fuels. The structure overcomes constrains in weak light harvesting and low electron–hole pair separation efficiency in the current nanostructured semiconductor catalyst materials. The 2‐nm‐thick TiO_2_ catalyst on a planar Al_2_O_3_ spacer and then on Al film resulted in a hydrocarbon formation rate of 0.967 mmol g^−1^ h^−1^, which is >1100 times higher than the commercial Aeroxide P25 photocatalyst. By integrating ultrathin TiO_2_ films in UFPLAs, hydrocarbon oxygenates, formic acid, and methanol, were also obtained with the optimized formation rate of 0.205 mmol g^−1^ h^−1^ from 2‐nm‐thick TiO_2_ film on 20 nm Al_2_O_3_ spacer in UFPLAs. The strong light harvesting capability and longer charge carrier lifetime are responsible for the catalyst structure activity. The surface defect, —OH group, Ti^3+^ species, VBM, etc., all greatly enhance the CO_2_ photoreduction to oxygenated hydrocarbons compared to nearly bare methane production from Aeroxide P25 catalyst. Moreover, it represents the record formation rate over the amount of photocatalysts in the family of titania photocatalysts to date. This discovery indicates the potential to improve thin‐film catalyst photocatalytic performance using nanophotonic‐managed light harvesting capabilities.

## 4 Experimental Section

*Modeling of the UFPLA*: The optical absorption of the UFPLA was modeled using COMSOL based on finite element method. In this modeling, the planar UFPLA was illuminated by the incident light within the wavelength range from 210 to 450 nm. By tuning the thickness of TiO_2_ layer, both the value and the spatial distribution of the optical absorption can be tuned effectively (e.g., as shown in Figure 1b,d, respectively). This numerical modeling helps to better understand how the UFPLA structure influences the optical properties and the consequent photocatalytic activities.

*UFPLA Preparation*: The 150‐nm‐thick Al films were deposited on glass substrates using Kurt J. Lesker AXXIS electron beam evaporator using the Al pellets (99.999%, EVMAL50QXQ‐D) purchased from Kurt J. Lesker. The pressure in the chamber during deposition was controlled at ≈5 × 10^−7^ Torr. A flow benchtop reactor (FlexAL, Oxford Instruments) was used to prepare the thin TiO_2_ and Al_2_O_3_ layers in the UFPLA via the ALD process. The ALD of Al_2_O_3_ was conducted by exposing the sample to trimethylaluminum (i.e., TMA, from SAFC Hitech) for 0.04 s, purging with Ar for 1.5 s, exposing the sample to deionized (DI) water for 3.5 s, and purging with Ar for 2.0 s again. The sample temperature was kept at 150 °C in the Al_2_O_3_ preparation process. The ALD of TiO_2_ was carried out by alternative exposure to titanium isopropoxide (i.e., TTIP, from Japan Advanced Chemicals) for 1.5 s and DI water at 200 °C for 3.5 s, followed by Ar purge for 1.5 s after each exposure. Each ALD cycle would result in 0.12‐nm‐thick Al_2_O_3_ or 0.05‐nm‐thick TiO_2_ deposition. For control experiment, the commercial titania (Aeroxide P25, titania ≥99.5% trace metal basis) was purchased from Sigma‐Aldrich and used as catalyst for the photocatalytic CO_2_ reduction.

*Catalyst Characterization*: The UFPLA structure was examined by the powder XRD using a Bruker D8 Advance Lynx Powder Diffractometer (LynxEye PSD detector, sealed tube, Cu Kα radiation with Ni β‐filter). The morphology of the samples after deposition of Al and TiO_2_ layers, respectively, was observed using the scanning electron microscopy (SEM, Zeiss CrossBeam Workstation system, see Figure S4, Supporting Information). The FTIR spectra of the samples were recorded with a spectrometer (Nicolet Magna‐IR 560) in the range of 400–4000 cm^−1^. Each sample was measured with 32 scans at an effective resolution of 2 cm^−1^. The surface composition of the UFPLAs was analyzed using a X‐ray photon spectrometer (Kratos AXIS 165) equipped with 165 mm radius hemispherical analyzer and eight channeltron detection system coupled with monochromatic Al radiation (1486.6 eV). The light absorption was measured using an Ocean Optics USB2000+ spectrometer equipped with an IS200‐4 integrating sphere detector, and the white high reflectance sphere material (manufactured from polytetrafluoroethylene (PTFE) based bulk material) was used as the reference. A spectroscopic ellipsometer (Horiba) was used to characterize the optical constants and thicknesses of Al_2_O_3_ and TiO_2_ layers in the UFPLAs.

*Transient Absorption Spectroscopy*: The time‐resolved experiments were conducted using ExciPro pump‐probe spectrometers (CDP). The fundamental output came from a Ti:sapphire femtosecond regenerative amplifier operating at 800 nm with 35 fs pulses and a 1 kHz repetition rate. The pump pulses at 365 nm were generated after passing through a fraction of the 800 nm beam into a spectrally tunable (450–910 nm) optical parametric amplifier (TOPAS Prime, Spectra‐Physics) and a frequency mixer (NirUVis, Light Conversion). The probe pulses (white light) were generated using another fraction of 800 nm—amplified pulses being focused onto 2‐mm‐thick sapphire plate contained in an Ultrafast System LLC spectrometer. The pump pulses were overlapped on the sample with the probe pulses after passing through a synchronized chopper (500 Hz), which blocked alternative pump pulses. Finally, the change in absorption (Δ*A*) of the excited state was calculated by subtracting the absorptions of the excited and unexcited samples. Detailed information about TA setup can be found elsewhere.^42^, ^43^

*Photocatalytic Reaction*: The photocatalytic CO_2_ reduction with water was run in a customized photocatalytic reactor, as shown in Figure S3 (Supporting Information). In the experiment, the UFPLA structure and 0.05 mL water were charged into the reactor and were treated in CO_2_ and Ar mixture at 42 and 9 mL min^−1^ for 50 min prior to the reaction. After the shut‐off valves located at inlet and outlet of the reactor were closed, the xenon (Xe) irradiation (OSRAM XBO 450W PFR) was applied onto the top window of the photocatalytic reactor. After 4 h irradiation, the Xe lamp was turned off and the gas mixture in the reactor was carried out by flowing CO_2_ at 3 mL min^−1^ to the gas chromatography for composition analysis. A 30 m × 0.25 mm capillary column (Supelco SP‐2330) and a 3.0 m × 3.18 mm packed column (Agilent HAYESEP DB), connected to a flame ionization detector and a thermal conductivity detector, respectively, were used to calibrate and separate the reactants and products. For comparison purpose, the photocatalytic CO_2_ reduction with water was run in the reactor without the presence of any catalyst and the commercial Aeroxide P25 catalyst, respectively.

## Conflict of Interest

The authors declare no conflict of interest.

## Supporting information

Supplementary

## References

[1] X. X. Chang , T. Wang , J. L. Gong , Energy Environ. Sci. 2016, 9, 2177.

[2] J. L. White , M. F. Baruch , J. E. Pander , Y. Hu , I. C. Fortmeyer , J. E. Park , T. Zhang , K. Liao , J. Gu , Y. Yan , T. W. Shaw , E. Abelev , A. B. Bocarsly , Chem. Rev. 2015, 115, 12888. 26444652 10.1021/acs.chemrev.5b00370

[3] Y. Zheng , W. Q. Zhang , Y. F. Li , J. Chen , B. Yu , J. C. Wang , L. Zhang , J. J. Zhang , Nano Energy 2017, 40, 512.

[4] T. Inoue , A. Fujishima , S. Konishi , K. Honda , Nature 1979, 277, 637.

[5] Y. C. Lan , Y. L. Lu , Z. F. Ren , Nano Energy 2013, 2, 1031.

[6] Y. Zhao , N. Hoivik , K. Y. Wang , Nano Energy 2016, 30, 728.

[7] B. B. Lakshmi , C. J. Patrissi , C. R. Martin , Chem. Mater. 1997, 9, 2544.

[8] G. K. Mor , K. Shankar , M. Paulose , O. K. Varghese , C. A. Grimes , Nano Lett. 2006, 6, 215. 16464037 10.1021/nl052099j

[9] H. G. Yang , C. H. Sun , S. Z. Qiao , J. Zou , G. Liu , S. C. Smith , H. M. Cheng , G. Q. Lu , Nature 2008, 453, 638. 18509440 10.1038/nature06964

[10] J. Schneider , M. Matsuoka , M. Takeuchi , J. L. Zhang , Y. Horiuchi , M. Anpo , D. W. Bahnemann , Chem. Rev. 2014, 114, 9919. 25234429 10.1021/cr5001892

[11] A. Dhakshinamoorthy , S. Navalon , A. Corma , H. Garcia , Energy Environ. Sci. 2012, 5, 9217.

[12] C. Aprile , A. Corma , H. Garcia , Phys. Chem. Chem. Phys. 2008, 10, 769. 18231679 10.1039/b712168g

[13] A. Fujishima , X. T. Zhang , D. A. Tryk , Surf. Sci. Rep. 2008, 63, 515.

[14] A. L. Linsebigler , G. Q. Lu , J. T. Yates , Chem. Rev. 1995, 95, 735.

[15] K. Mori , H. Yamashita , M. Anpo , RSC Adv. 2012, 2, 3165.

[16] M. A. Kats , R. Blanchard , P. Genevet , F. Capasso , Nat. Mater. 2013, 12, 20. 23064496 10.1038/nmat3443

[17] H. Dotan , O. Kfir , E. Sharlin , O. Blank , M. Gross , I. Dumchin , G. Ankonina , A. Rothschild , Nat. Mater. 2013, 12, 158. 23142836 10.1038/nmat3477

[18] H. Song , L. Guo , Z. Liu , K. Liu , X. Zeng , D. Ji , N. Zhang , H. Hu , S. Jiang , Q. Gan , Adv. Mater. 2014, 26, 2737. 24616090 10.1002/adma.201305793

[19] C. Janisch , H. Song , C. Zhou , Z. Lin , A. L. Elías , D. Ji , M. Terrones , Q. Gan , Z. Liu , 2D Mater. 2016, 3, 025017.

[20] Z. Xia , H. Song , M. Kim , M. Zhou , T. H. Chang , D. Liu , X. Yin , K. Xiong , H. Mi , X. Wang , F. Xia , Z. Yu , Z. J. Ma , Q. Gan , Sci. Adv. 2017, 3, e1602783. 28695202 10.1126/sciadv.1602783 PMC5501504

[21] C. G. Morales‐Guio , S. D. Tilley , H. Vrubel , M. Gratzel , X. Hu , Nat. Commun. 2014, 5, 3059. 24402352 10.1038/ncomms4059

[22] A. Paracchino , V. Laporte , K. Sivula , M. Gratzel , E. Thimsen , Nat. Mater. 2011, 10, 456. 21552270 10.1038/nmat3017

[23] I. A. Digdaya , G. W. P. Adhyaksa , B. J. Trzesniewski , E. C. Garnett , W. A. Smith , Nat. Commun. 2017, 8, 15968. 28660883 10.1038/ncomms15968 PMC5493770

[24] M. R. Nellist , F. A. L. Laskowski , J. J. Qiu , H. Hajibabaei , K. Sivula , T. W. Hamann , S. W. Boettcher , Nat. Energy 2018, 3, 46.

[25] L. J. Liu , Y. Li , Aerosol Air Qual. Res. 2014, 14, 453.

[26] K. F. Li , X. Q. An , K. H. Park , M. Khraisheh , J. W. Tang , Catal. Today 2014, 224, 3.

[27] T. L. Lopez , J. A. Moreno , R. Gomez , X. Bokhimi , J. A. Wang , H. Yee‐Madeira , G. Pecchi , P. Reyes , J. Mater. Chem. 2002, 12, 714.

[28] H. Jensen , A. Soloviev , Z. S. Li , E. G. Sogaard , Appl. Surf. Sci. 2005, 246, 239.

[29] J. Meyer , H. Schmidt , W. Kowalsky , T. Riedl , A. Kahn , Appl. Phys. Lett. 2010, 96, 243308.

[30] L. H. Kim , K. Kim , S. Park , Y. J. Jeong , H. Kim , D. S. Chung , S. H. Kim , C. E. Park , ACS Appl. Mater. Interfaces 2014, 6, 6731. 24712401 10.1021/am500458d

[31] K. L. Pickrahn , A. Garg , S. F. Bent , ACS Catal. 2015, 5, 1609.

[32] L. B. Xiong , J. L. Li , B. Yang , Y. Yu , J. Nanomater. 2012, 2012, 831524.

[33] I. Bertoti , M. Mohai , J. L. Sullivan , S. O. Saied , Appl. Surf. Sci. 1995, 84, 357.

[34] M. M. Rahman , K. M. Krishna , T. Soga , T. Jimbo , M. Umeno , J. Phys. Chem. Solids 1999, 60, 201.

[35] K. Ikeue , H. Yamashita , M. Anpo , T. Takewaki , J. Phys. Chem. B﻿﻿ 2001, 105, 8350.

[36] K. Ikeue , S. Nozaki , M. Ogawa , M. Anpo , Catal. Lett. 2002, 80, 111.

[37] P. Salvador , J. Appl. Phys. 1984, 55, 2977.

[38] Y. Tamaki , A. Furube , M. Murai , K. Hara , R. Katoh , M. Tachiya , Phys. Chem. Chem. Phys. 2007, 9, 1453. 17356752 10.1039/b617552j

[39] J. Y. Sun , Y. Yang , J. I. Khan , E. Alarousu , Z. B. Guo , X. X. Zhang , Q. Zhang , O. F. Mohammed , ACS Appl. Mater. Interfaces 2014, 6, 10022. 24918499 10.1021/am5026159

[40] H. Song , S. Jiang , D. Ji , X. Zeng , N. Zhang , K. Liu , C. Wang , Y. Xu , Q. Gan , Opt. Express 2015, 23, 7120. 25837057 10.1364/OE.23.007120

[41] S. Qamar , F. C. Lei , L. Liang , S. Gao , K. T. Liu , Y. F. Sun , W. X. Ni , Y. Xie , Nano Energy 2016, 26, 692.

[42] A. O. El‐Ballouli , E. Alarousu , M. Bernardi , S. M. Aly , A. P. Lagrow , O. M. Bakr , O. F. Mohammed , J. Am. Chem. Soc. 2014, 136, 6952. 24521255 10.1021/ja413254g

[43] R. Begum , M. R. Parida , A. L. Abdelhady , B. Murali , N. M. Alyami , G. H. Ahmed , M. N. Hedhili , O. M. Bakr , O. F. Mohammed , J. Am. Chem. Soc. 2017, 139, 731. 27977176 10.1021/jacs.6b09575

