# Supplementary material for: Ultrathin‐Film Titania Photocatalyst on Nanocavity for CO2 Reduction with Boosted Catalytic Efficiencies
Source: Glob Chall. 2018 Sep 19;2(11):1800032. doi: 10.1002/gch2.201800032 (PMC6282774; doi:10.1002/gch2.201800032)
Supplement: Supplementary file 1 — Supplementary [file GCH2-2-1800032-s001.pdf]

Copyright WILEY-VCH Verlag GmbH & Co. KGaA, 69469 Weinheim, Germany, 2018.

Global Challenges
Open Access

Supporting Information

for *Global Challenges,* DOI: 10.1002/gch2.201800032

Ultrathin-Film Titania Photocatalyst on Nanocavity for CO_2 Reduction with Boosted Catalytic Efficiencies

Haomin Song, Wei Wu, Jian-Wei Liang, Partha Maity, Yuying Shu, Nam Sun Wang, Omar F. Mohammed, Boon S. Ooi, Qiaoqiang Gan, and Dongxia Liu**

Supplementary material

Ultra-thin film Titania photocatalyst on nanocavity for CO_2 reduction with boosted catalytic efficiencies

Haomin Song[a,1], Wei Wu[b,1], Jian-Wei Liang[c], Partha Maity[d], Yuying Shu[e], Nam Sun Wang[b], Omar F. Mohammed[d], Boon S. Ooi[c], Qiaoqiang Gan[a,*], Dongxia Liu[b,*]

[a] *Department of Electrical Engineering, The State University of New York at Buffalo, Buffalo, NY 14260*

[b] *Department of Chemical & Biomolecular Engineering, University of Maryland College Park, MD 20742*

[c] *Department of Electrical Engineering, Photonics Lab, King Abdullah University of Science and Technology, Thuwal, Saudi Arabia*

[d] *Department of Material Science, King Abdullah University of Science and Technology, Thuwal, Saudi Arabia*

[e] *W. R. Grace and Company, 7500 Grace Dr, Columbia, MD 21044, USA*

[1] These two authors contribute equally to this work.
* Corresponding authors: qqgan@buffalo.edu; liud@umd.edu

S1. Optical constants of ultra-thin TiO_2 and Al_2O_3 films

To better design the UFPLA with higher absorption, the optical constants of TiO_2 and Al_2O_3 films were characterized using a spectroscopic ellipsometry (Horiba), as shown in Figure S1. The refractive index, *n*, and extinction coefficient, *k*, are presented by blue solid curves and red dashed curves, respectively.

a b

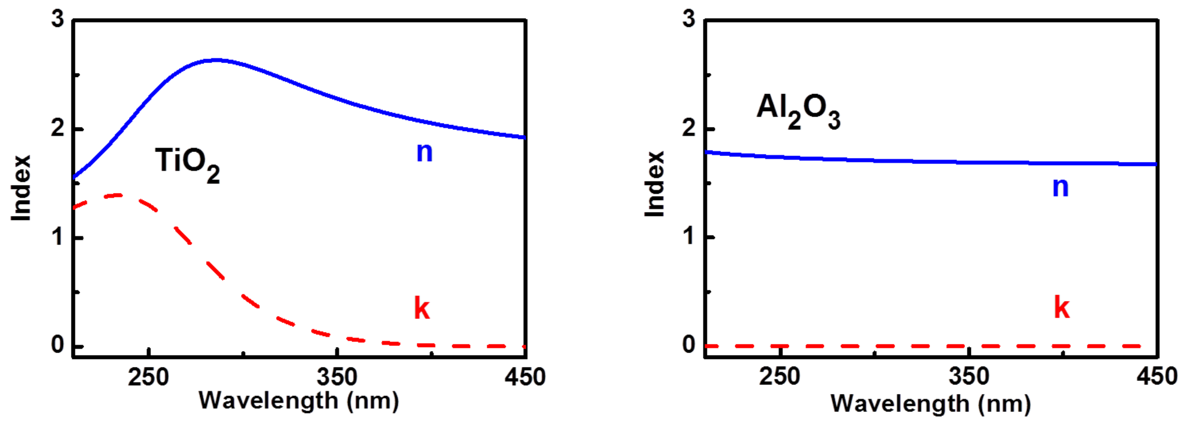

Fig. S1. The optical constants of ultra-thin (a) TiO_2 and (b) Al_2O_3 films.

S2. The original absorption spectra of Fig. 1c

To obtain a color contour similar to Fig. 1c, we first measure the absorption spectra of the five samples with different TiO_2 film thickness. The original experimental data is shown in Fig. S2.

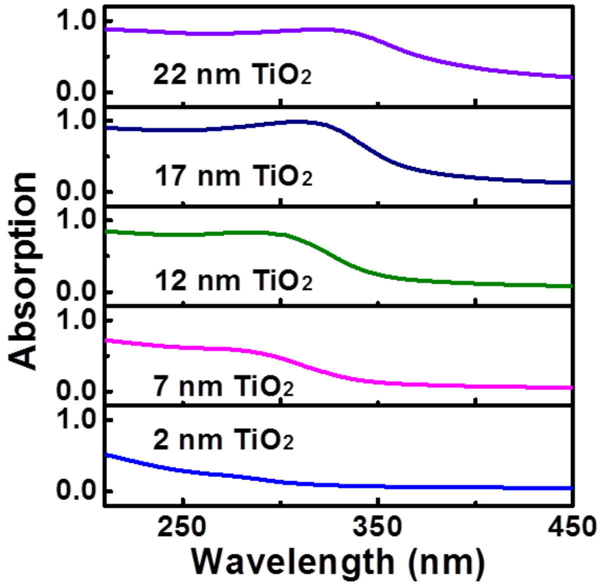

Fig. S2. The original absorption spectra of Fig. 1c, showing the measured total absorption spectra of TiO_2/Al samples with different TiO_2 film thickness.

S3. Experimental setup for photocatalytic reaction

The photocatalytic CO_2 reduction with water was run in a customized photocatalytic reactor. **Fig. S3a** shows the schematic diagram of the experimental set-up. The experimental apparatus consisted mainly of two gas cylinders (Argon (Ar) and CO_2, respectively), two mass flow controllers (AALBORG, GFCS model), a photocatalytic reactor, and a gas chromatography instrument (Agilent Technologies, 6890N). The reactor (**Fig. S3b**) consists of a stainless steel body and two quartz windows of 5.0 cm in diameter and 0.5 cm in thickness. The internal volume of the reactor is 14.1 mL. Two gas connection lines (inlet and outlet of the reactor) stay opposite to each other on the perimeter wall of the cylindrical photocatalytic reactor body.

a

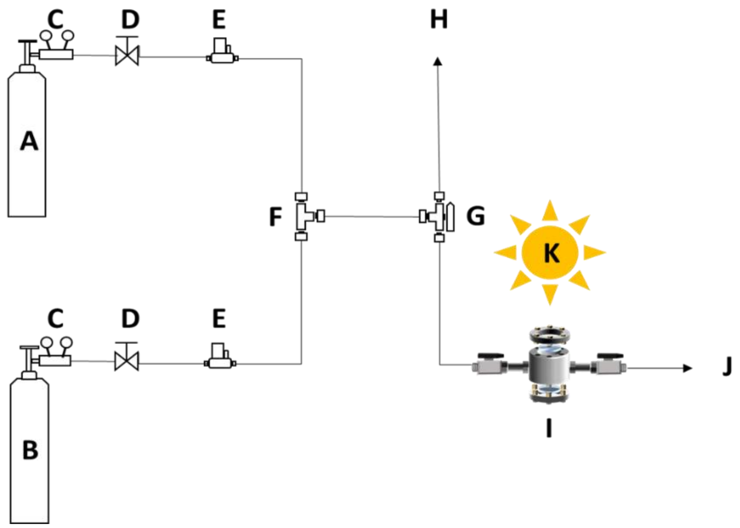

b

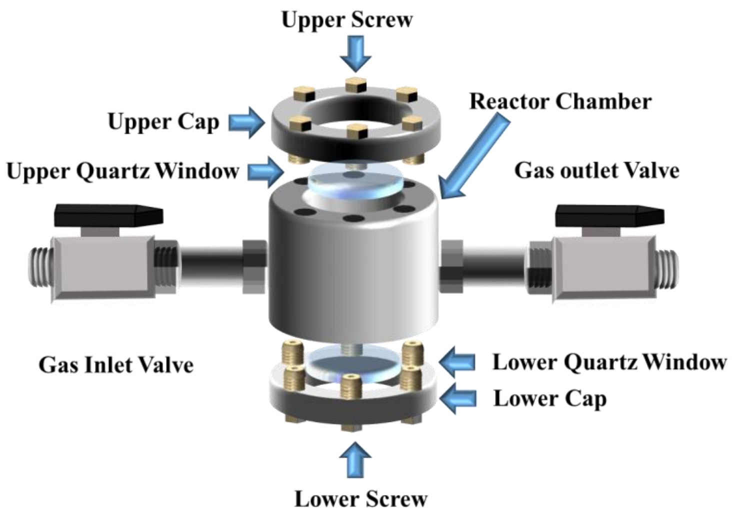

Fig. S3. Schematic diagram of the reaction system (a) [A: Argon cylinder, B: CO_2 cylinder, C: cylinder regulator, D: shut-off valve, E: mass flow controller, F: union tee connector, G: 3-way valve, H: flowmeter, I: photocatalytic reactor, J: gas chromatography instrument, K: xenon lamp] and structure of the photocatalytic reactor cell (b) used for photocatalytic carbon dioxide reduction with water.

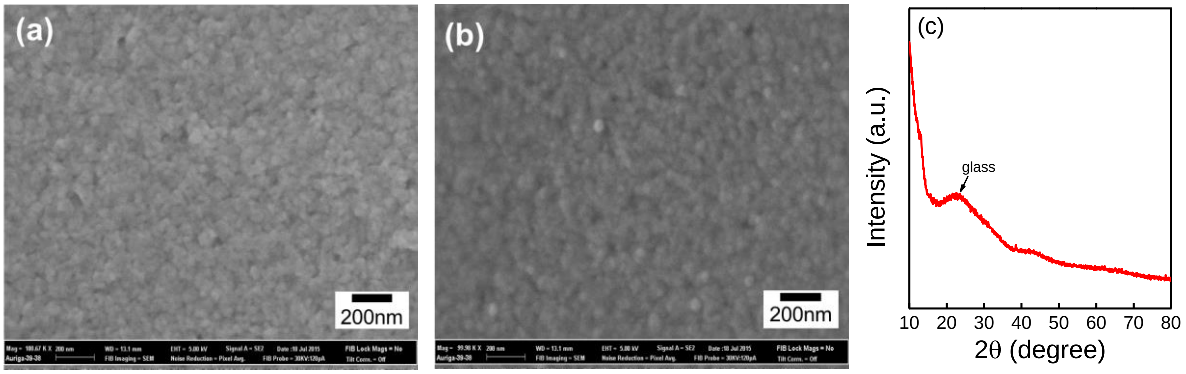

Fig. S4. SEM showing morphology of (a) Al layer deposited onto flat glass substrate and (b) TiO_2 layer deposited onto Al layer on the glass substrate. (c) shows XRD spectrum of the TiO_2/Al/glass UFPLA structure. The diffraction peak is assigned to the glass substrate [S1].

S4. Mass of TiO_2 film catalyst in the UFPLA

Table S1. Mass of TiO_2 film catalyst in the UFPLA structure.

**Two-layered Al/TiO_2 cavity system**					
Sample name	2-nm-TiO_2/Al	7-nm-TiO_2/Al	12-nm-TiO_2/Al	17-nm-TiO_2/Al	22-nm-TiO_2/Al
TiO_2 thickness (nm)	2	7	12	17	22
TiO_2 mass (g)	1.69×10^{-6}	5.92×10^{-6}	1.02×10^{-5}	1.44×10^{-5}	1.86×10^{-5}

**Three-layered nanocavity with 2-nm-TiO_2 films**							
Sample name	2-nm -TiO_2/ 5-nm -Al_2O_3 /Al	2-nm -TiO_2/ 10-nm -Al_2O_3 /Al	2-nm -TiO_2/ 15-nm -Al_2O_3 /Al	2-nm -TiO_2/ 20-nm -Al_2O_3 /Al	2-nm -TiO_2/ 25-nm -Al_2O_3 /Al	2-nm -TiO_2/ 30-nm -Al_2O_3 /Al	2-nm -TiO_2/ 35-nm -Al_2O_3 /Al
TiO_2 thickness (nm)	2	2	2	2	2	2	2
TiO_2 mass (g)	1.69×10^{-6}	1.69×10^{-6}	1.69×10^{-6}	1.69×10^{-6}	1.69×10^{-6}	1.69×10^{-6}	1.69×10^{-6}

The mass of TiO_2 film catalyst in each UFPLA was calculated in order to evaluate the specific photocatalytic activity that is presented in Fig. 2b. The geometrical surface area of TiO_2 film is 2 cm^2 since we purposely controlled all UFPLAs to have the same geometrical surface area in the fabrication process. The density of TiO_2 is assumed to be 4.23 g cm^{-3}.[S2] The mass of TiO_2 film equals to the product of the film thickness, the surface area (i.e., 2cm^2) and the density (i.e., 4.23 g cm^{-3}). **Table S1** lists the catalyst amount of each TiO_2 film in the UFPLA.

S5. Detailed sample introduction procedure

To avoid any contamination, the reactor was taken down, washed with DI water and dried by flowing dry air before each experiment. After lower parts assembling, shown in **Fig. S3b**, the P25 or UFPLA was placed at the top of lower quartz window, then 0.05 g DI water was added into the reactor chamber to function as the oxidant in the reaction. The upper parts were then sealed. CO_2 and Ar mixture was flowed through the reactor in dark condition for 50 min to eliminate the air entrapped during the reactor assembly and catalyst loading processes, at 42-mL min^{-1} and 9-mL min^{-1} flow rate, respectively. GC was used, at this time, to assert the completeness in removal of entrapped air in the reactor chamber. Finally, CO_2 and Argon gas flows were stopped, two valves right next to the reactor body were shut off, and the Xe light was turned on to start the reaction.

References

[S1] R. Chakraborty, A. Dey, A. K. Mukhopadhyay, *Metall. Mater. Trans. A* **2010**, *41a*, 1301.
[S2] N. Rahimi, R. A. Pax, E. M. Gray, *Prog. Solid State Chem.* **2016**, *44*, 86.
